# Bidirectional submucosal tunneling endoscopic resection for a subepithelial tumor at the gastric cardia

**DOI:** 10.1055/a-2922-5376

**Published:** 2026-08-05

**Authors:** Naoya Tada, Noriko Nishiyama, Ryosuke Kawanishi, Kaho Nakatani, Joji Tani, Takayoshi Kishino, Hideki Kobara

**Affiliations:** 1 Department of Gastroenterology and Neurology, Faculty of Medicine 12850 Kagawa UniversityTakamatsuKagawaJapan; 2 Nishiyama Memorial Medical Corporation, MIRAI HospitalSakaideKagawaJapan; 3 Department of Gastroenterological Surgery, Faculty of MedicineKagawa UniversityTakamatsuKagawaJapan

**Fig. 1:**
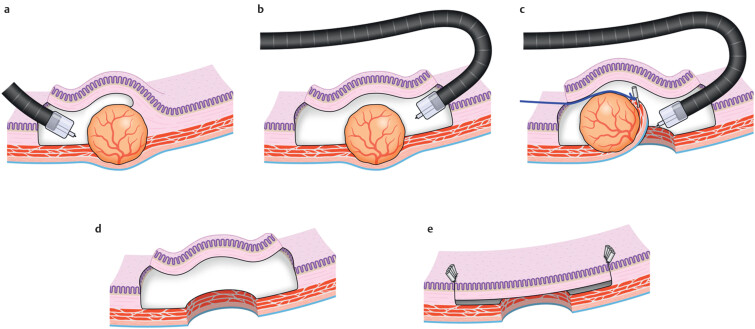
A schematic diagram of the bidirectional submucosal tunneling endoscopic resection. ( **a** ) Submucosal tunnel creation from the proximal side. ( **b** ) Submucosal tunnel creation from the distal side. ( **c** ) Incision of the muscle layer using clip-line traction. ( **d** ) Complete resection. ( **e** ) Closure of the mucosal incision sites.

Submucosal tunneling endoscopic resection (STER) is an established treatment for upper gastrointestinal submucosal tumors originating from the muscularis propria. 1​ ​2​ However, STER is inherently limited. The narrow tunnel distal to the tumor restricts endoscope maneuverability, increasing tumor injury risk during resection, leading to lower complete resection rates. 2​ ​3​ Furthermore, full-thickness defect closure at the cardia remains technically challenging. 4​ To address these, we developed a bidirectional approach (Bi-STER; Fig. 1 , Video 1 ).

**Video 1** Complete resection of a gastric cardia subepithelial tumor by bidirectional submucosal tunneling endoscopic resection (Bi-STER), followed by closure of the mucosal entry sites.


**Fig. 2:**
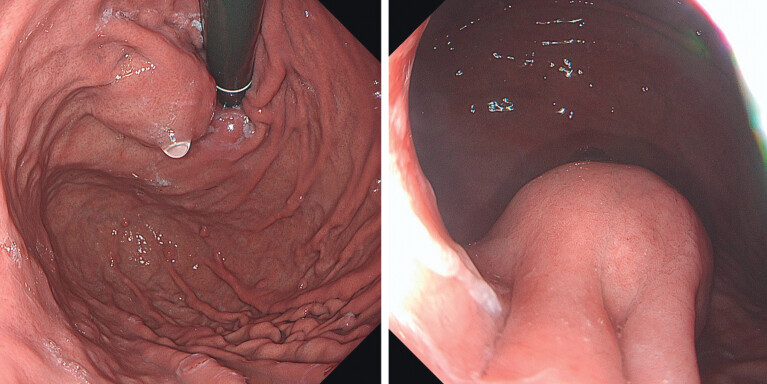
A 25-mm subepithelial tumor at the gastric cardia.

**Fig. 3:**
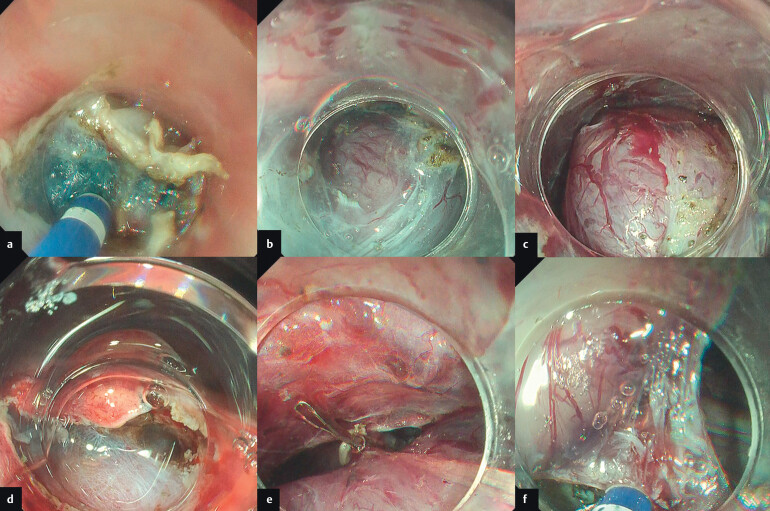
Procedural steps of bidirectional submucosal tunneling endoscopic resection. ( **a** ) Mucosal incision and submucosal entry approximately 3 cm proximal to the tumor under forward-view endoscopy. ( **b** ) Proximal submucosal tunnel toward the tumor. ( **c** ) Muscular layer exposure at the tumor attachment and submucosal dissection over the tumor apex and along both lateral sides. ( **d** ) Second mucosal incision and submucosal entry approximately 3 cm distal to the tumor, followed by creation of a distal submucosal tunnel under retroflexion from the anal side. ( **e** ) Clip-with-line application to the distal side of the tumor within the tunnel to retract it orally. ( **f** ) Incision of the muscular layer surrounding the tumor from a bidirectional view.

**Fig. 4:**
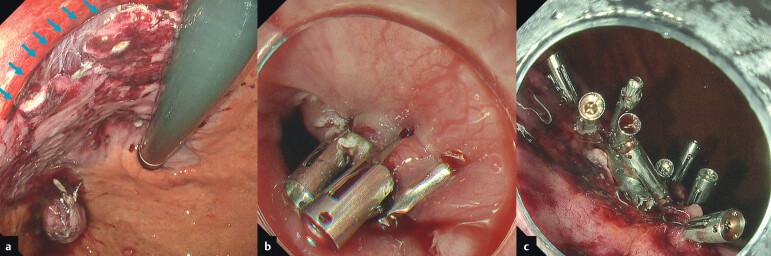
( **a** ) After bidirectional submucosal tunneling endoscopic resection, with blue arrows pointing at the mucosal entry site of the distal side. ( **b** and **c** ) Both the proximal and distal mucosal entry sites were completely closed using endoscopic clips ( **b** : proximal side in the esophagus and **c** : distal side in the stomach).

**Fig. 5:**
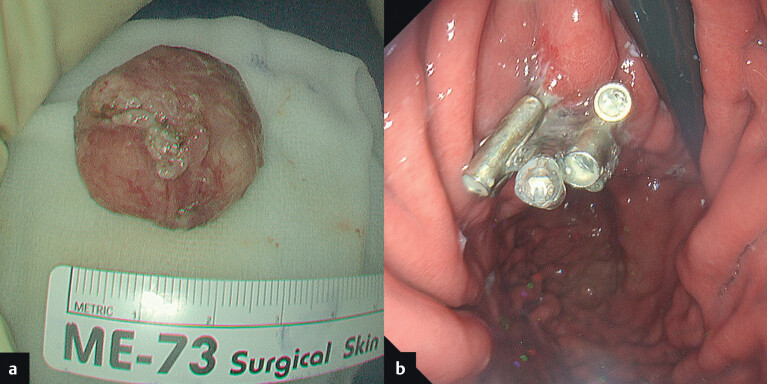
( **a** ) Resected subepithelial tumor measuring 25 mm. ( **a** ) 2 months after the bidirectional submucosal tunneling endoscopic resection.

A 70-year-old man presented with a 25-mm subepithelial tumor at the gastric cardia ( Fig. 2 ). After written informed consent was obtained, Bi-STER was performed as follows ( Fig. 3 ). First, under forward-view endoscopy, a mucosal incision was made approximately 3 cm proximal to the tumor, and a submucosal tunnel was created toward the tumor. The muscular layer at the tumor attachment was exposed, and submucosal dissection was done over the tumor apex and along both lateral sides. Under retroflex-view endoscopy from the anal side, a second mucosal incision from the distal side was made approximately 3 cm distal to the tumor, and a second submucosal tunnel was created. After muscular layer exposure and tumor apex and lateral side dissection, a clip-with-line was applied to the distal side of the tumor within the tunnel to retract the tumor orally, facilitating distal dissection and preventing intraperitoneal migration. Muscular layer incision surrounding the tumor attachment was performed under countertraction, achieving en bloc enucleation. The tumor was delivered into the gastric lumen and retrieved orally ( Fig. 4 ). Both mucosal entry sites were closed with clips ( Fig. 5 ). The postoperative course was uneventful. Histology confirmed a very-low-risk gastrointestinal stromal tumor (modified Fletcher classification) with tumor-free margins.

Bi-STER, combining proximal forward-view tunneling and distal retroflex tunneling, enabled the circumferential visualization of the tumor attachment, with easy closing of mucosal incisions and safe and complete en bloc resection without tumor injury.

Endoscopy_UCTN_Code_CPL_1AH_2AZ_3AZ
